# Therapeutic Notes

**Published:** 1900-06

**Authors:** 


### Therapeutic Notes.

### Mnscular Rheumatism.

℞ Sodii salicylatis.
Potass. acetrtis aa..................dr. 4
Glycerini..........................oz. 2
Acquæ q. s. ad...................oz. 4

M. Sig. One teaspoonful in one-half glass of water or milk every two hours.—*Ex.*

### Children's Emetic.

℞ Pulv. ipecacuan...............gr. viiss

Antimonii et potassi tartratis... gr. $\frac{1}{6}$

Oxymel scillæ................ ʒiiss

Aq. distill. q. s. ad........... ℥i

M. S. One teaspoonful every ten minutes.—*Baginsky.*

### Inhalation in Tuberculous Laryngitis.

℞ Menthol.

Ether. sulphuric.

Ol. pini sylvestris.

Tinct. iodi aa.......................ʒij

Tinct. benzoin. co. ad.............℥ij

M. S. Ten drops on an oro-nasal inhaler which is worn as much of the time as possible.— *W. Fowler.*

### Treatment of Baldness.

Dr. Whitla gives this as one of the best combinations in the treatment of baldness:

℞ Pilocarp. hydrochloratis..........gr. 5
Otto rosæ.........................m. 8
Ol. rosmarini.....................dr. 4
Linimenti cantharidis.............dr. 4
Glycerini puri............-......oz. 1
Ol. amygdalæ dulcis..............oz. 3
Spts. Camphoræ.................oz. 3

M. Sig. To be rubbdd well into the scslp night and morning. —*Ex.*

